# Amino Acid Metabolites Associated with Chronic Kidney Disease: An Eight-Year Follow-Up Korean Epidemiology Study

**DOI:** 10.3390/biomedicines8070222

**Published:** 2020-07-17

**Authors:** Hansongyi Lee, Han Byul Jang, Min-Gyu Yoo, Sang Ick Park, Hye-Ja Lee

**Affiliations:** Center for Biomedical Sciences, Korea National Institute of Health, Cheongju 28159, Korea; flowerlhsy@korea.kr (H.L.); greatstar@korea.kr (H.B.J.); yoomingku@korea.kr (M.-G.Y.); parksi@nih.go.kr (S.I.P.)

**Keywords:** amino acid, chronic kidney disease, incidence, inflammation, metabolites

## Abstract

The discovery of metabolomics-based biomarkers has been a focus of recent kidney dysfunction research. In the present study, we aimed to identify metabolites associated with chronic kidney disease (CKD) in the general population using a cross-sectional study design. At baseline, 6.5% of subjects had CKD. Pearson correlation analysis showed that 28 metabolites were significantly associated with estimated glomerular filtration rate (eGFR) after Bonferroni correction. Among these metabolites, 4 acylcarnitines, 12 amino acids, 4 biogenic amines, 1 phosphatidylcholine, and 1 sphingolipid were associated with CKD (*p* < 0.05). After eight years, 13.5% of subjects had CKD. Three amino acid metabolites were positively associated with new-onset CKD: citrulline [odds ratio (OR): 2.41, 95% confidence interval (CI): 1.26–4.59], kynurenine (OR: 1.98, 95% CI: 1.05–3.73), and phenylalanine (OR: 2.68, 95% CI: 1.00–7.16). The kynurenine:tryptophan ratio was also associated with CKD (OR: 3.20; 95% CI: 1.57–6.51). The addition of multiple metabolites significantly improved the CKD prediction by C statistics (0.756–0.85, *p* < 0.0001), and the net reclassification improvement was 0.84 (95% CI: 0.72–0.96). Elevated hs-C reactive protein (CRP) was associated with new-onset CKD (OR: 1.045, 95% CI: 1.005–1.086); however, this association disappeared following adjustment with the kynurenine:tryptophan ratio. The levels of citrulline and kynurenine and their ratio to tryptophan in CKD patients with proteinuria were worse than those with one or neither characteristic. Together, the results of this study demonstrate that amino acid metabolites are associated with CKD eight years after initial metabolite assessment. These results could improve the identification of subjects at high risk of CKD who have modified amino acid metabolism.

## 1. Introduction

More than 11% of the global population suffers from chronic kidney disease (CKD) [1]. CKD results in gradual loss of kidney function leading to end-stage renal disease, requiring dialysis or renal transplantation [2]. Early-stage CKD has few signs or symptoms, such that the disease is often not detected until the later stages; however, the risk of cardiovascular mortality and morbidity increases with CKD progression [3,4]. It is therefore important to identify a predictive biomarker for CKD in the general population.

CKD biomarker research has focused on metabolomics-based discovery. The kidney directly impacts circulating metabolite levels, including their uptake by glomerular filtration, tubular secretion, and catabolism and the release of several amino acids and other metabolites [5,6,7]. Recent studies of CKD have applied metabolite profiling to a longitudinal setting [8,9,10]. In the Atherosclerosis Risk in Communities (ARIC) cohort study, two novel metabolites (5-oxoproline and 1-,5-anhydroglucitol) were found to be related to significantly reduced CKD incidence in a 19.6-year follow-up study [8]. In the Framingham Heart Study (FHS), nine metabolites were associated with significantly increased CKD incidence after eight years [9]. In the Cooperative Health Research in the Region of Augsburg (KORA) study, the kynurenine:tryptophan ratio was associated with CKD incidence after seven years [10]. Furthermore, a decline in kidney function can be accompanied by changes in metabolite concentrations [11,12], and metabolites have been reported to predict kidney dysfunction [13,14,15].

To our knowledge, the association between metabolites and CKD incidence in Korea has not been intensively investigated. Therefore, we identified the plasma metabolites related to the estimated glomerular filtration rate (eGFR) and CKD using data from the Korean Genome and Epidemiology Study (KoGES). We further identified selected metabolites as CKD biomarker candidates according to their association with CKD eight years after the initial study.

## 2. Methods

### 2.1. Study Population

**Figure 1:**
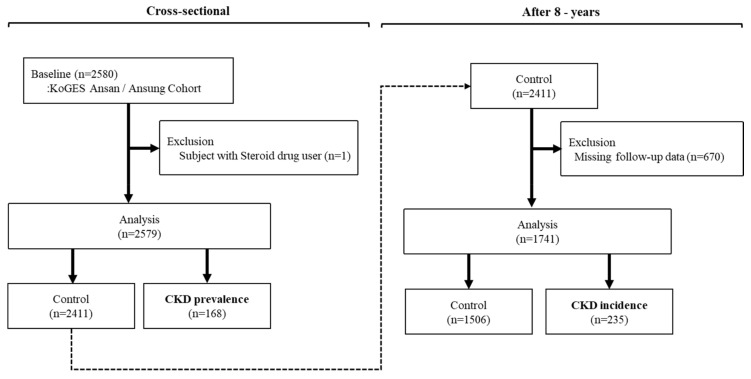
Flow chart of the study population.

This study used data drawn from the second follow-up of the Ansan–Ansung population cohort study (2005–2006) in the Korean Genome Epidemiology Study [16]. Subjects with metabolite information at baseline (*n* = 2580) were included. Among these, one steroid user was excluded (*n* = 1), as were subjects with missing creatinine data at baseline (*n* = 337). Thus, we analyzed a total of 2579 subjects in the cross-sectional study. To examine the CKD incidence after eight years, we excluded 168 CKD patients at baseline and subjects with missing creatinine data at follow-up (*n* = 680). A total of 1741 subjects were therefore analyzed (Figure 1). The study protocol was approved by the Institutional Review Board of the Korea Centers for Disease Control and Prevention (nos. 2017-02-06 and 2017-02-07).

### 2.2. Definitions of eGFR and CKD

Kidney function was evaluated based on eGFR using the CKD-Epidemiology Collaboration equation, which considered race, age, sex, and serum creatinine concentration [17]:

GFR = 141 (female: 144) × min (Scr/ҡ, 1)^α^ × max (Scr/ҡ, 1)^–1^.^209^ × 0.993^age^ × 1.018 (if female) (1)

where ҡ is 0.7 and 0.9 for female and male subjects, respectively; α is –0.320 and –0.411 for female and male subjects, respectively; min indicates the minimum of Scr/ҡ or 1; and max indicates the maximum of Scr/ҡ or 1. CKD was defined as an eGFR of < 60 mL/min/1.73 m^2^.

### 2.3. General and Blood Parameters

General characteristics (age, gender, drinking status, smoking status, and comorbidities) and anthropometrics (height, weight, and body mass index) of the subjects were investigated. Data on systolic and diastolic blood pressure, blood parameters related to kidney status (blood urea nitrogen, creatinine, and proteinuria), glucose, hemoglobin A1_C_ (HbA1_C_), and high-sensitivity C-reactive protein (hs-CRP) were obtained from the KoGES database. Proteinuria was defined as the presence of protein > grade 1 using the urine dipstick test.

### 2.4. Metabolite Level Measurements

Serum metabolite data were obtained from the KoGES database, and assays were conducted according to the manufacturer’s instructions [18]. Briefly, serum samples were collected from the 2580 subjects at baseline. Liquid chromatography and flow injection analysis mass spectrometry were performed using the AbsoluteIDQ p180 kit (Biocrates Life Sciences, Innsbruck, Austria). A total of 134 metabolites were analyzed, including 13 acylcarnitines, 21 amino acids, 9 biogenic amines, 34 phosphatidylcholine diacyls (PCs aa C), 36 phosphatidylcholine acyl-alkyls (PCs ae C), 8 lyso PCs, 12 sphingomyelins, and 1 hexose.

### 2.5. Statistical Analyses

Comparisons of subject characteristics between groups were assessed using the chi-squared test for categorical variables or the Wilcoxon rank sum test for continuous variables. In subsequent analyses, variables with non-normal distributions (e.g., metabolites and hs-CRP) were log-transformed prior to analysis. To screen for eGFR-related metabolites, correlations between metabolite levels and eGFR were assessed using Pearson’s partial correlation coefficients, with Bonferroni correction (*p* < 0.00037). Multivariable logistic regression analysis was performed to estimate OR values for CKD prevalence and incidence in association with the screened metabolites. To examine the predictive ability of multiple amino acid metabolites (citrulline, kynurenine, phenylalanine, and kynurenine:tryptophan) for CKD, we performed C statistics-based model discrimination. To ensure that the model could correctly reclassify risk groups, we assessed net reclassification improvement, and to test the ability of the model to evaluate increased average sensitivity, we assessed integrated discrimination improvement. Differences in metabolite levels among the four groups (patients with CKD and/or proteinuria) were assessed using a general linear model adjusted for age, sex, BMI, smoking, drinking, systolic blood pressure, HbA_1_C, and hs-CRP. Statistical analyses were performed using the SAS ver. 9.4 software (SAS Institute, Inc., Cary, NC). Statistical significance was assessed at a level of *p* < 0.05.

## 3. Results

### 3.1. Baseline Subject Characteristics

Table 1 summarizes the baseline characteristics of subjects with and without CKD. Among all subjects, 6.5% had CKD at baseline. The proportion of subjects exhibiting current drinking was higher in the control group than the CKD group, whereas the prevalence of hypertension and diabetes was higher in the CKD group than the control group (*p* < 0.05). The mean systolic blood pressure was higher in subjects with CKD than in those without, after the data were adjusted for age, sex, drinking, smoking, and the prevalence of hypertension and diabetes mellitus (*p* < 0.05).

### 3.2. Correlations between Metabolites and eGFR

Table 2 shows the 28 metabolites that were found to be significantly associated with eGFR after Bonferroni correction. Five acylcarnitines, 13 amino acids, 4 biogenic amines, 1 phosphatidylcholine, and 4 sphingolipids were negatively correlated with eGFR, and phosphatidylcholine was positively correlated with eGFR after adjustment for age, sex, energy, body mass index, smoking status, drinking status, systolic blood pressure, HbA1C, and hs-CRP.

### 3.3. Metabolites Associated with CKD

Among the candidate metabolites, 4 acylcarnitines, 12 amino acids, 4 biogenic amines, 1 phosphatidylcholine, and 1 sphingolipid were positively associated with CKD (Table 3). Kynurenine had the highest OR for CKD (OR: 13.81; 95% CI: 7.38–25.86), followed by citrulline (OR: 10.42; 95% CI: 5.34–20.14), methionine (OR: 5.35; 95% CI: 2.78–10.29), arginine (OR: 4.94; 95% CI: 2.66–9.20), C4 (OR: 4.76; 95% CI: 3.06–7.40), phenylalanine (OR: 4.33; 95% CI: 1.57–11.88), alanine (OR: 4.37; 95% CI: 2.06–9.29), isoleucine (OR: 4.29; 95% CI: 2.00–9.19), leucine (OR: 3.83; 95% CI: 1.65–8.92), and asparagine (OR: 3.75; 95% CI: 1.72–8.15). The kynurenine:tryptophan (OR: 12.65; 95% CI: 6.55–24.44), phenylalanine:tyrosine (OR: 5.65; 95% CI: 1.88–17.00), and glycine:serine ratios (OR: 12.37; 95% CI: 4.79–31.96) were also associated with CKD.

### 3.4. Baseline Characteristics of Subjects with and without CKD after Eight Years

Among the 1741 subjects, 13.5% were found to have CKD in our eight-year follow-up study. The proportion of subjects who reported current drinking was higher for the control group than for the CKD group, whereas the prevalence of hypertension and diabetes was higher in the CKD group (*p* < 0.05). Mean body mass index, HbA1C, and hs-CRP values were significantly higher in the CKD group than in the control group after adjusting for age, sex, drinking, smoking, and the prevalence of hypertension and diabetes (*p* < 0.05) (Table 4).

### 3.5. Amino Acid Metabolites as Predictive Markers of CKD

Using a cross-sectional design, we selected 22 metabolites associated with CKD prevalence (Table 3) and performed multivariate logistic regression analysis for CKD incidence after eight years. Three amino acid metabolites showed a positive association with CKD, after adjusting for age, sex, BMI, smoking status, drinking status, systolic blood pressure, HbA1C, proteinuria, and eGFR (baseline):citrulline (OR: 2.41; 95% CI: 1.26–4.59), kynurenine (OR: 1.98; 95% CI: 1.05–3.73), and phenylalanine (OR: 2.68; 95% CI: 1.00–7.16) (Table 5). The kynurenine:tryptophan ratio was also associated with CKD (OR: 3.20; 95% CI: 1.57–6.51). The addition of the multiple amino acid metabolites (citrulline, kynurenine, phenylalanine, and kynurenine:tryptophan) to C statistics of the CKD prediction showed an improvement in discrimination (0.756–0.85; *p* < 0.0001) (Table 6). Multiple amino acid metabolites showed significant improvement in classification accuracy, with a net reclassification improvement of 0.84 (95% CI: 0.72–0.96) and integrated discrimination improvement of 0.12 (95% CI: 0.10–0.14).

### 3.6. Association between Baseline hs-CRP and CKD Incidence Among Different Metabolites

We also confirmed that the association between baseline hs-CRP level and CKD after eight years was dependent on metabolites (Table 7). Baseline hs-CRP was positively associated with CKD (OR: 1.045; 95% CI: 1.005–1.086) after adjustment for age, sex, body mass index, smoking status, drinking status, systolic blood pressure, HbA1c, eGFR (baseline), and proteinuria (Model 1). This association was still significant after additional adjustment with kynurenine (OR: 1.042; 95% CI: 1.003–1.083), whereas that after adjustment with the kynurenine:tryptophan ratio (OR: 1.09; 95% CI: 0.97–1.21) was not (Model 2).

### 3.7. Comparison of Metabolite Concentration in Subjects with and without CKD or Proteinuria

**Figure 2:**
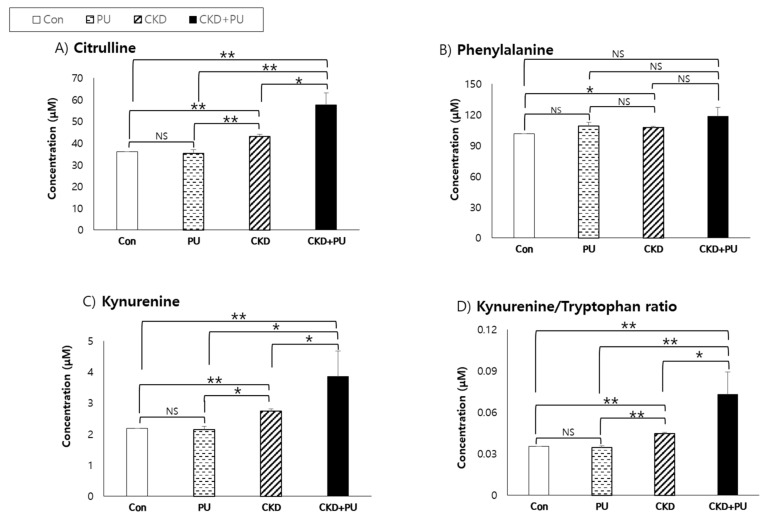
Comparison of metabolite concentrations between subjects with and without CKD or proteinuria. CKD, chronic kidney disease; Con, control; PU, proteinuria. Levels of metabolites (**A**: citrulline, **B**: kynurenine, **C**: phenylalanine, and **D**: kynurenine:tryptophan ratio) were log-transformed prior to analysis. * *p* < 0.05; ** *p* < 0.0001, adjusted for age, sex, body mass index, smoking status, drinking status, systolic blood pressure, HbA1C, and hs-CRP. NS: no significance between groups.

Baseline citrulline levels, as well as kynurenine and kynurenine:tryptophan levels, were significantly higher in patients with both CKD and proteinuria than in those with one or neither condition (*p* < 0.05) (Figure 2). Levels of these metabolites tended to be higher in subjects with CKD than in those with proteinuria alone. However, there were no differences in citrulline, kynurenine, or kynurenine:tryptophan levels between patients without both (control) and those with only proteinuria. In contrast, phenylalanine levels were significantly different between the CKD and control groups, and there were no differences between the control, proteinuria, and CKD + proteinuria groups.

## 4. Discussion

This is the first prospective study to investigate the association between plasma metabolites and CKD in the Korean population. Using data from an eight-year follow-up cohort study, we found 22 metabolites (4 acylcarnitines, 12 amino acids, 4 biogenic amines, 1 phosphatidylcholine, and 1 sphingolipid) related to CKD prevalence as well as baseline eGFR. Among these metabolites, citrulline, kynurenine, and the kynurenine:tryptophan ratio were found to be associated with a risk of developing CKD.

Early prediction of reduced kidney function would assist prevention of CKD and its complications. Thus, it is important to find biomarkers that can detect decreased kidney function early within the general population. In the present study, we found 28 metabolites cross-sectionally correlated with eGFR, compared with previous studies (Table S1) [9,10,19,20] and confirmed that most of these were related to clinically relevant outcomes of CKD (eGFR < 60 mL/min/1.73 m^2^). The KORA study, which had a larger sample size, also performed metabolomics analysis using a 150p kit similar to that used in this study, and determined that acylcarnitines were strongly associated with decreased eGFR [20]. Since acylcarnitines are excreted by the glomerulus, decreased eGFR may reduce acylcarnitine emissions, thereby increasing the concentration of acylcarnitines in the blood [21]. Long-chain acylcarnitines are involved in fatty acid metabolism, whereas short- or medium-chain acylcarnitines may be more closely associated with amino acid metabolism [22]. Similarly, we observed consistent association with reduced eGFR among four short- or medium-chain acylcarnitines (C3, C4, C7-DC, and C8). We also identified 12 amino acid metabolites that were associated with reduced eGFR. Amino acid metabolites may be linked to changes in kidney function via enzymatic activity (e.g., conversion of glycine to serine), oxidative stress (e.g., conversion of methionine sulfoxide to methionine), or inhibition of endothelial nitric oxide synthesis (e.g., conversion of citrulline to arginine). In our study, single metabolites including branched-chain amino acids, alanine, arginine, asparagine, citrulline, glycine, histidine, methionine, phenylalanine, proline, and their ratios (phenylalnine:tyrosine and glycine:serine) were cross-sectionally associated with reduced eGFR. However, some amino acid metabolites related to oxidative stress (methionine sulfoxide/methionine) or nitric oxide production (citrullin/arginine), which were previously reported to be associated with CKD, were not found to be associated with a decline in kidney function in this study [23,24].

To determine whether the cross-sectional association between these metabolites and eGFR is a result of decreased kidney function or whether they play a biological role in CKD pathogenesis, it is important to assess these relationships over time. In the present study, we assessed the association between 22 baseline metabolites and CKD incidence after eight years in subjects without CKD at baseline, and found that only two amino acid metabolites, citrulline and kynurenine, affected the development of CKD. Citrulline is generated from ornithine and carbamoyl phosphate in the urea cycle [25] and from arginine via a reaction catalyzed by nitric oxide synthase [26]. L-arginine is first oxidized into N-hydroxyl-arginine, which is further oxidized to citrulline with the release of nitric oxide [27]. In this study, citrulline showed an association with both CKD prevalence and CKD incidence after eight years, whereas the citrulline:arginine ratio showed no association with CKD. Our results are similar to those of previous studies involving patients with CKD stages 3–5, in which reduced kidney function was associated with increased plasma and urine citrulline concentrations [11,12]. In the FHS, citrulline was found to be strongly associated with CKD (OR: 1.48; CI: 1.19–1.83), although metabolite ratios were not evaluated [9]. Therefore, high-concentration citrulline is promising for its significant association with CKD incidence.

One of the metabolites examined in this study showed significant association with CKD; kynurenine was reported in a previous longitudinal study as an indicator for predicting CKD [9]. Kynurenine is the degradation product of tryptophan by hepatic tryptophan 2, 3-dioxygenase (TDO) and extrahepatic indoleamine 2, 3- dioxygenase (IDO) and is excreted in urine after being taken up from blood by the kidneys [28,29]. Thus, reduced kidney function is associated with the retention of kynurenine and its metabolites, because the kidney is the primary organ responsible for the elimination of kynurenine and its derivatives [30,31]. A previous animal study also demonstrated that kynurenine accumulation might be caused by accelerated synthesis and reduced metabolism [25]. In the present study, the kynurenine:tryptophan ratio, which is known to reflect IDO activity, was strongly associated with CKD incidence, which supports the previous report that kynurenine:tryptophan is a predictor of CKD [10]. Another study that analyzed tryptophan metabolism in patients with CKD reported that increased IDO activity was observed in an early stage (stage 2) of CKD [32]. These results suggest that upregulation of IDO-mediated tryptophan metabolism may be an early feature of CKD.

Because IDO is potently induced by proinflammatory cytokines [33,34], tryptophan metabolism is increased in the chronic inflammatory state. Similarly, our data demonstrated that hs-CRP, an inflammatory marker, is negatively correlated with tryptophan and positively correlated with kynurenine and its ratio (data not shown; tryptophan, r = –0.09; kynurenine, r = 0.09; kynurenine:tryptophan, r = 0.17; all *p* < 0.0001). Previous studies have reported that elevation of most inflammatory markers including hs-CRP, TNF-αR2, WBC count, and IL6 can predict the risk of developing CKD [35,36,37]. Although we did not measure other inflammatory markers, our results expand these earlier findings by demonstrating that CKD progression due to an increase in hs-CRP may be partially mediated by IDO activity. This finding is consistent with the observation that the induction of IDO activity in CKD patients may primarily be a consequence of chronic inflammation [38]. These findings suggest that kynurenine and its ratio to tryptophan have a partial role in the pathophysiology of CKD, which is induced by inflammatory factors.

Phenylalanine, an indispensable amino acid, is converted to tyrosine, a semi-essential amino acid, which is synthesized only by the phenylalanine 4-hydroxylase enzyme [39,40]. In chronic renal failure in humans and rats, plasma and renal phenylalanine remain normal or are slightly increased, tyrosine concentrations in plasma and skeletal muscle are often decreased, and the phenylalanine:tyrosine ratio in plasma and muscle is slightly increased [41,42,43,44]. As a result of impairment in the conversion of phenylalanine to tyrosine in chronic renal failure, tyrosine and its ratio to phenylalanine are reduced in plasma and many tissues. In the present study, we confirmed that phenylalanine was negatively correlated with reduced renal function and could be a useful predictor of CKD incidence and prevalence, perhaps due to reduced activity of the phenylalanine 4-hydroxylase enzyme and reduced tyrosine levels in the CKD group.

Since several studies have reported that proteinuria is an independent marker of risk for adverse CKD outcomes [45,46], we further analyzed whether subjects with both reduced eGFR and proteinuria have higher levels of theses metabolites than those with one or neither condition. We found that subjects with both low eGFR and proteinuria had worse citrulline, kynurenine, and kynurenine:tryptophan levels than those with one or neither condition, suggesting that high levels of these metabolites well reflect the high-risk group for adverse CKD outcomes. A prior study examined only metabolites related to proteinuria in CKD and found that 4-hydroxycholrthalonil, 1,5-anhydroglucitol and metabolites of the phosphatidylethanolamine pathway were strongly associated with proteinuria in subjects with CKD [47]. Although we did not analyze these metabolites in the present study due to the small number of subjects having both low eGFR and proteinuria (*n* = 12), we observed that proteinuria alone was more closely related to acylcarnitines and phosphatidylcholine than to amino acid metabolites (Table S2). These results indicate that the pathogenesis of proteinuria and decreased GFR may be caused by different mechanisms. Further studies are needed to understand these mechanisms and to establish the metabolites of high-risk subjects with both proteinuria and reduced GFR.

This study had some common limitations. GFR was estimated by a single creatinine measurement, although eGFR is the gold standard diagnostic method for CKD. We also performed a nonglobal analysis, which limited the number of metabolites that could be evaluated. Additionally, we did not adjust for protein or amino acid intake or urine albumin in the multivariable logistic regression model, because these data were not available. Further research is needed to explore the global metabolites related to kidney function, confirmation of CKD status, and direct enzyme activities integrated with amino acid metabolites. Nevertheless, this study had a large sample size and applied long-term follow-up of the association between metabolites and CKD in the Korean population.

## 5. Conclusions

In conclusion, we observed that high concentrations of baseline amino acids and their ratios were associated with CKD incidence in an eight-year follow-up study. Multiple amino acid metabolites showed higher predictive ability than single metabolites. We determined that amino acid levels and their ratios differed according to inflammation or proteinuria status. Thus, amino acids and their ratios, as underlying drivers of CKD pathology, can be used as biomarkers of CKD due to their modified metabolism. Absolute quantification of amino acid metabolites and their regulatory enzyme activities is needed to design CKD treatment strategies.

## Figures and Tables

**Table 1:** Baseline characteristics of subjects with and without chronic kidney disease (CKD).

	Control	CKD	*p* ^†^	*p* ^††^
Participants (%)	2411 (93.5)	168 (6.5)	–	–
Age (years)	56.5 ± 8.9	65.2 ± 6.8	<0.0001	–
Sex (%)			0.0983	–
Male	1149 (47.7)	69 (41.1)		
Female	1262 (52.3)	99 (58.9)		
Drinking status (%)			<0.0001	–
Never	1124 (46.4)	101 (60.1)		
Former	123 (5.1)	16 (9.5)		
Current	1163 (48.3)	51 (30.4)		
Smoking status (%)			0.3250	–
Never	1472 (61.1)	104 (61.9)		
Former	437 (18.1)	36 (21.4)		
Current	501 (20.8)	28 (16.7)		
Proteinuria			<0.0001	
Urine protein < 1	2368 (98.4)	156 (92.9)		
Urine protein ≥ 1+	39 (1.6)	12 (7.1)		
Comorbidities				
Hypertension (%)	810 (33.6)	102 (60.7)	<0.0001	–
Diabetes mellitus (%)	533 (22.2)	55 (32.7)	0.0016	–
Body mass index (kg/m^2^)	24.5 ± 3.2	25.1 ± 3.4	0.0538	0.1130
Systolic blood pressure (mm Hg)	117.8 ± 16.7	122.0 ± 16.9	0.0009	0.0042
Diastolic blood pressure (mm Hg)	78.6 ± 10.3	78.9 ± 9.5	0.5560	0.0088
Blood urea nitrogen (mg/dL)	15.5 ± 4.1	18.4 ± 5.5	<0.0001	<0.0001
Creatinine (mg/dL)	0.96 ± 0.14	1.25 ± 0.49	<0.0001	<0.0001
e-GFR (mL/min/1.73 m^2^)	79.3 ± 11.0	53.0 ± 7.2	<0.0001	<0.0001
Glucose (mg/dL)	95.7 ± 19.3	97.7 ± 27.5	0.3983	0.5775
HbA1_C_ (%)	5.7 ± 0.8	5.9 ± 0.7	<0.0001	0.8804
Hs-CRP (mg/L)	1.6 ± 3.5	2.3 ± 5.9	0.0003	0.4572

e-GFR, estimated glomerular filtration rate; Hb A1_C,_ hemoglobin A1_C_; Hs-CRP, high-sensitivity C-reactive protein. ^†^ Data are presented as number (%) or mean ± standard deviation (SD), compared using the chi-squared test for categorical variables and the Mann–Whitney U test for continuous variables. ^††^ Adjusted for age, sex, drinking status, smoking status, hypertension, and diabetes mellitus (*p* < 0.05).

**Table 2:** Relationship between metabolites and estimated glomerular filtration rate (eGFR).

Metabolite	*r* *	*p*
C3 (Propionylcarnitine)	–0.10	<0.0001
C4 (Butyrylcarnitine)	–0.20	<0.0001
C7-DC (Pimelylcarnitine)	–0.10	<0.0001
C8 (Octanoylcarnitine)	–0.11	<0.0001
C14:2 (Tetradecadienylcarnitine)	–0.09	<0.0001
Alanine	–0.10	<0.0001
Arginine	–0.12	<0.0001
Asparagine	–0.10	<0.0001
Citrulline	–0.21	<0.0001
Glutamine	–0.09	<0.0001
Glycine	–0.11	<0.0001
Histidine	–0.08	<0.0001
Isoleucine	–0.13	<0.0001
Leucine	–0.10	<0.0001
Methionine	–0.10	<0.0001
Phenylalanine	–0.09	<0.0001
Proline	–0.12	<0.0001
Valine	–0.09	<0.0001
Acetylornithine	–0.16	<0.0001
Kynurenine	–0.24	<0.0001
Putrescine	–0.09	<0.0001
Sarcosine	–0.09	<0.0001
PCaaC28:1	–0.07	0.0003
PCaaC42:5	0.08	0.0001
SMOHC14:1	–0.09	<0.0001
SMOHC16:1	–0.08	<0.0001
SMOHC22:2	–0.08	<0.0001
SMC18:1	–0.08	<0.0001

PC aa, phosphatidylcholine diacyl; SM, sphingomyelin; SMOH, hydroxysphingomyelin. * Partial correlation coefficients adjusted for age, sex, body mass index, smoking status, drinking status, systolic blood pressure, HbA_1_C, and hs-CRP.

**Table 3:** Association (odds ratio, OR) and 95% confidence intervals (CIs) between metabolites and CKD prevalence.

Metabolites	OR (CIs) ^†^
C3 (Propionylcarnitine)	3.41 (2.08–5.60)
C4 (Butyrylcarnitine)	4.76 (3.06–7.40)
C7-DC (Pimelylcarnitine)	1.94 (1.27–2.97)
C8 (Octanoylcarnitine)	1.92 (1.26–2.91)
C14:2 (Tetradecadienylcarnitine)	1.39 (0.99–1.95)
Alanine	4.37 (2.06–9.29)
Arginine	4.94 (2.66–9.20)
Asparagine	3.75 (1.72–8.15)
Citrulline	10.42 (5.34–20.14)
Glutamine	2.20 (0.99–4.87)
Glycine	2.56 (1.25–5.23)
Histidine	3.23 (1.37–7.62)
Isoleucine	4.29 (2.00–9.19)
Leucine	3.83 (1.65–8.92)
Methionine	5.35 (2.78–10.29)
Phenylalanine	4.33 (1.57–11.88)
Proline	3.71 (2.08–6.60)
Valine	2.75 (1.08–7.01)
Acetylornithine	2.38 (1.77–3.20)
Kynurenine	13.81 (7.38–25.86)
Putrescine	1.37 (1.05–1.78)
Sarcosine	1.70 (1.19–2.44)
PCaaC28:1	2.16 (1.12–4.16)
PCaaC40:5	0.98 (0.64–1.52)
SMOHC14:1	1.35 (0.69–2.65)
SMOHC16:1	1.23 (0.65–2.33)
SMOHC22:2	1.53 (0.77–3.04)
SMC18:1	2.09 (1.07–4.08)
Citrulline/Arginine	1.41 (0.88–2.27)
Glycine/Serine	12.37 (4.79–31.96)
Phenylalanine/Tyrosine	5.65 (1.88–17.00)
Kynurenine/Tryptophan	12.65 (6.55–24.44)

PC aa, phosphatidylcholine diacyl; SM, sphingomyelin; SMOH, hydroxysphingomyelin. ^†^ Multivariate logistic regression adjusted for age, sex, body mass index, smoking status, drinking status, systolic blood pressure, HbA_1_C, and hs-CRP.

**Table 4:** Baseline characteristics of subjects with and without CKD after eight years.

	Control	CKD Incidence	*p* ^†^	*p* ^††^
Participants (%)	1506 (86.5)	235 (13.5)	–	–
Age (years)	54.8 ± 8.3	62.3 ± 7.6	<0.0001	–
Sex (%)			0.1338	–
Male	707 (47.0)	98 (41.7)		
Female	799 (53.0)	137 (58.3)		
Drinking status (%)			0.0009	–
Never	698 (45.1)	135 (57.5)		
Former	61 (4.1)	14 (6.0)		
Current	747 (49.6)	86 (36.6)		
Smoking status (%)			0.2660	–
Never	945 (62.8)	160 (68.1)		
Former	270 (17.9)	38 (16.2)		
Current	291 (19.3)	37 (15.7)		
Proteinuria			<0.0001	
Urine protein < 1	1484 (99.1%)	223 (95.7%)		
Urine protein ≥ 1+	13 (0.9%)	10 (4.3%)		
Comorbidities				
Hypertension (%)	449 (29.8)	111 (47.2)	<0.0001	–
Diabetes mellitus (%)	297 (19.8)	53 (22.6)	0.3208	–
Body mass index (kg/m^2^)	24.6 ± 3.1	25.1 ± 3.6	0.0372	0.0274
Systolic blood pressure (mm Hg)	116.6 ± 15.9	121.4 ± 16.8	<0.0001	0.7207
Diastolic blood pressure (mm Hg)	78.4 ± 10.1	79.5 ± 10.1	0.1843	0.8209
Blood urea nitrogen (mg/dL)	15.4 ± 4.0	16.3 ± 3.9	0.0002	0.1366
Creatinine (mg/dL)	0.95 ± 0.14	0.99 ± 0.14	0.0008	<0.0001
e-GFR (mL/min/1.73 m^2^)	91.1 ± 14.2	85.3 ± 13.2	<0.0001	<0.0001
Glucose (mg/dL)	95.4 ± 18.7	96.1 ± 18.9	0.4086	0.7544
Hemoglobin A1_C_ (%)	5.7 ± 0.7	5.9 ± 0.8	<0.0001	0.0023
Hs-CRP (mg/L)	1.5 ± 2.6	2.5 ± 6.6	<0.0001	0.0033

e-GFR, estimated glomerular filtration rate; Hb A1_C_, hemoglobin A1_C_; Hs-CRP, high-sensitivity C-reactive protein. ^†^ Data are presented as number (%) or mean ± standard deviation, compared using the chi-squared test for categorical variables and Mann–Whitney U test for continuous variables. ^††^ Adjusted for age, sex, drinking status, smoking status, hypertension, and diabetes mellitus (*p* < 0.05).

**Table 5:** Association between metabolites and CKD incidence after eight years.

Metabolites	OR (95% CI) ^†^
Citrulline	2.41 (1.26–4.59)
Kynurenine	1.98 (1.05–3.73)
Phenylalanine	2.68 (1.00–7.16)
Kynurenine:tryptophan	3.20 (1.57–6.51)

^†^ Multivariate logistic regression after adjustment for age, sex, body mass index, smoking status, drinking status, systolic blood pressure, HbA_1_C, eGFR (baseline), and proteinuria.

**Table 6:** Multiple amino acid metabolites and prediction of CKD incidence.

Variables	Multiple Amino Acid Metabolites ^†^	C Statistics		*p*
		Base Model	Base Model + Metabolites	
OR per SD	1.06 (1.04–1.09)	0.76	0.85	<0.0001 ^††^
Net reclassification improvement (Category-free)	0.84 (0.72–0.96)			<0.0001
Integrated discrimination improvement	0.12 (0.10–0.14)			<0.0001

^†^ Citrulline, kynurenine, phenylalanine, and kynurenine:tryptophan. ^††^ Base model vs. Base model + metabolites. Values in parentheses are 95% CIs.

**Table 7:** Association between baseline hs-CRP and CKD incidence according to metabolites.

	OR (95% CI)
Model 1 ^†^	1.045 (1.005–1.086)
Model 2 ^††^	
Adj. Model 1 + kynurenine	1.042 (1.003–1.083)
Adj. Model 1 + kynurenine:tryptophan	1.033 (0.996–1.073)

^†^ Model 1: multivariate logistic regression after adjustment for age, sex, body mass index, smoking status, drinking status, systolic blood pressure, HbA1c, eGFR (baseline), and proteinuria. ^††^ Model 2: multivariate logistic regression after adjustment for model 1 + metabolites.

## Supplementary Materials

Supplementary materials can be found at https://www.mdpi.com/2227-9059/8/7/222/s1.
