# Supplementary material for: Amino Acid Metabolites Associated with Chronic Kidney Disease: An Eight-Year Follow-Up Korean Epidemiology Study
Source: Biomedicines. 2020 Jul 17;8(7):222. doi: 10.3390/biomedicines8070222 (PMC7399801; doi:10.3390/biomedicines8070222)
Supplement: Supplementary file 1 [file biomedicines-08-00222-s001.pdf]

Amino Acid Metabolites Associated with Chronic Kidney Disease: An Eight-Year Follow-Up Korean Epidemiology Study

Hansongyi Lee, Han Byul Jang, Min-Gyu Yoo, Sang Ick Park and Hye-Ja Lee

Table S1. Comparison of 28 metabolites associated with kidney function in the present study and previous studies.

Metabolite	CRIC [19]	FSH [9]	KORA S4 [10]	KORA F4 [20]	TwinsUK [20]	Meta [20,*]
C3 (Propionylcarinitine)	NS	-	NS	○	○	○
C4 (Butyrylcarnitine)	NS	-	NS	○	○	○
C7-DC (Pimelylcarnitine)	NS	-	NS	○	○	○
C8 (Octanoylcarnitine)	NS	-	NS	○	○	○
C14:2 (Tetradecadienylcarnitine)	-	-	NS	NS	○	○
Alanine	NS	-	○	-	-	-
Arginine	○	-	○	○	-	-
Asparagine	NS	-	NS	-	-	-
Citrulline	NS	○	NS	-	-	-
Glutamine	NS	-	○	○	-	-
Glycine	NS	-	○	NS	NS	○
Histidine	NS	-	NS	NS	NS	-
Isoleucine	NS	-	NS	-	-	-
Leucine	NS	-	NS	-	-	-
Methionine	○	-	NS	NS	NS	-
Phenylalanine	○	-	NS	NS	NS	○
Proline	NS	-	○	NS	NS	-
Valine	NS	-	NS	NS	NS	-
Acetylornithine		-	○	-	-	-
Kynurenine	NS	○	○	-	-	-
Putrescine		-	-	-	-	-
Sarcosine	NS	-	-	-	-	-
PCaaC28:1	-	-	NS	NS	-	-
PCaaC42:5	-	-	○	NS	-	-
SMOHC14:1	-	-	NS	NS	-	-
SMOHC16:1	-	-	NS	NS	-	-
SMOHC22:2	-	-	NS	NS	-	-
SMC18:1	-	-	NS	○	-	-

PC aa, phosphatidylcholine diacyl; SM, sphingomyelin; SMOH, hydroxysphingomyelin. CRIC, Chronic Renal Insufficiency Cohort Study; FSH, Framingham Heart study; KORA, Cooperative Health Research in the Region of Augsburg. ○: associated with kidney function, NS: non-significant ($p > 0.05$) −: did not analysis in this study. [*]Meta-analysis using the KORA F4 and TwinsUK sample.

Table S2. Association (OR and 95% CI) between metabolites and proteinuria prevalence.

Metabolites	OR (CIs) †
C2 (Acetylcarnitine)	6.56 (2.87-14.99)
C7-DC (Pimelylcarnitine)	2.17 (1.01-4.65)
C8 (Octanoylcarnitine)	2.11 (1.01-4.40)
C14:1 (Tetradecenoylcarnitine)	3.98 (1.75-9.06)
C14:2 (Tetradecadienylcarnitine)	1.97 (1.04-3.73)
C18:0 (Octadecanoylcarnitine)	3.76 (1.38-10.27)
Glutamate	2.23 (1.07-4.68)
Putrescine	2.12 (1.26-3.56)
Sarcosine	2.23 (1.50-3.30)
Spermine	0.19 (0.07-0.54)
PCaaC28:1	6.38 (2.00-20.34)
PCaaC30:0	2.86 (1.22-6.72)
PCaaC32:0	4.03 (1.48-11.00)
PCaaC32:1	2.04 (1.18-3.53)
PCaaC34:1	3.84 (1.56-9.45)
PCaaC34:2	3.85 (1.27-11.71)
PCaaC36:1	2.85 (1.13-7.14)
PCaaC36:2	3.53 (1.16-10.79)
PCaeC34:1	4.61 (1.43-14.85)
PCaeC36:0	2.29 (1.04-5.06)
PCaeC36:1	4.58 (1.38-15.23)
PCaeC38:1	2.24 (1.09-4.65)

† Multivariate logistic regression after adjustment for age, sex, body mass index, smoking status, drinking status, systolic blood pressure, HbA_1C, hs-CRP, and eGFR (baseline).
